# Maternal Body Mass Index Trends and Weight Gain in Singleton Pregnancies at the Time of Fetal Anatomic Survey: Changes in the Last Decade and New Trends in the Modern Era

**DOI:** 10.3390/nu15224788

**Published:** 2023-11-15

**Authors:** Alexandra Ursache, Iuliana Elena Bujor, Alexandra Elena Cristofor, Denisa Oana Zelinschi, Dragos Nemescu, Daniela Roxana Matasariu

**Affiliations:** 1 Department of Obstetrics and Gynecology, University of Medicine and Pharmacy ‘Gr. T. Popa’, 700115 Iasi, Romania; alexandra.ursache@umfiasi.ro (A.U.); iuliana-elena.bujor@d.umfiasi.ro (I.E.B.); zelinschi.denisa@email.umfiasi.ro (D.O.Z.); daniela.matasariu@umfiasi.ro (D.R.M.); 2 Department of Obstetrics and Gynecology, Cuza Voda Hospital, 700038 Iasi, Romania

**Keywords:** pregnancy, obesity, epidemiology, first trimester morphology, second trimester morphology, overweight

## Abstract

(1) Background: the worldwide impact of overweight and obesity is rising, increasingly resembling an epidemic (a price we have to pay for our new way of living). (2) Methods: our study aims to evaluate the temporal trends and patterns of singleton pregnant women’s BMI (body mass index) in our region during a 12-year time frame between 2010 and 2021. (3) Results: We noticed a statistically significant difference between the BMIs of nulliparous and multiparous women and a significantly increased pregestational BMI in women with previous ART (assisted reproductive technology) procedures. Smoking pregnant women had a higher second trimester weight gain, regardless of parity. Women with folic acid supplementation alone had a higher BMI than those with folic acid and multivitamin intake. The weight of both nulliparous and multiparous women with chronic hypertension was statistically significantly higher in all three timeframes. Global weight gain did not reveal any statistically significant changes concerning women with pregestational diabetes, regardless of parity and the pregnancy trimester. (4) Conclusions: our article describes the trends in obesity and overweight in our middle-income country, in which this pathology is continuously growing, negatively influencing our reproductive-aged women and future generations.

## 1. Introduction

Overweight and obesity have increased during the past few decades. The worldwide impact is rising, increasingly resembling an epidemic (a price we have to pay for our new way of living) [1,2]. Urbanization-related sedentariness associated with processed low-quality food negatively influences our health, resulting in a continuously increasing number of obese and overweight people. This has become a major public health issue, affecting not only the populations of developed countries but also those of low- and middle-income countries [3]. Our healthcare systems need to reshape the increasing pathologies associated with overweight and obesity [4]. In their retrospective study about women’s healthcare utilization and costs, Morgan et al. revealed an increase of 23% in overweight pregnancies and 37% in obese pregnancies [5].

This condition has affected many women, and the curve still has an upward slope [6]. In some countries, the rates of obesity exceed 30%. This ascending trend has also become visible in Asian countries. In China, the rates have tripled since 2004 [3]. The rates of overweight and obesity among European women reached up to 44.7% in the last decade [2,7]. A national study in the United States revealed that almost 50% of pregnant women are either overweight or obese [8].

Overweight and obesity are defined using World Health Organization (WHO) criteria. BMI (body mass index) is calculated by dividing an individual’s weight in kilograms by the square of their height in meters. After obtaining their BMI value, we can classify the individual as being underweight with a BMI under 18.5; normal weight with a BMI that ranges from 18.5 to 24.9; overweight with a BMI from 25 to 29.9; or obese with a BMI above 30. Obesity is also classified into three stages depending on the BMI value: class I (BMI = 30.0–34.9), class II (35.0–39.9), or class III (≥40.0) [9].

Nutritional disorders negatively impact both mothers’ and children’s outcomes [10]. Obesity and overweight are associated with almost all pregnancy complications, affecting women before conception, during pregnancy, and postpartum. In the pregestational period, both overweight and obesity cause fertility problems, miscarriages, chronic hypertension, type 2 diabetes, stroke, and heart problems. Women who start their pregnancy with a weight above normal and a BMI over 24.9 are more prone to placental (abnormal spiral arterial modification and placental hypertrophy), embryonic, and fetal growth pathologies. Overweight and obese women have an increased risk of birth complications, including cesarean delivery, instrumental delivery, induction of labor, obstructed labor, shoulder dystocia, hypertensive disorders, gestational diabetes, preterm births, thromboembolisms, stillbirths, infections, birth defects, large-for-gestational-age (LGA) fetuses, and macrosomia. They are also have an increased risk of postpartum complications, including hemorrhages, infections, and thromboembolisms [1,2,7,10,11,12,13]. The impact on the offspring is reflected in an increase in mortality and morbidity in fetuses and newborn children, and, as some studies depict, in long-term negative consequences in descendants that extend beyond the gestational period. As the literature reveals, children from obese mothers develop hypertensive disorders, diabetes, obesity, and cardiovascular dysfunctions [14] due to an inadequate intrauterine environment [15]. In the end, we cannot neglect or deny the economic implications of nutritional disorders, with both overweight and obesity indicating the prolonged need to address healthcare services [6,11]. These overwhelming complications of obesity do not improve by simply reducing weight gain during pregnancy due to pre-existing fat that is responsible for all the negative results stated above. Some studies have reported that the sources of all negative maternal and fetal outcomes are directly linked to the mother’s weight before the pregnancy and less to weight gain during it [6].

The literature provides scarce data on weight trends in the obstetric population. In our country, only one study (conducted by Panaitescu et al.) has evaluated the prevalence of underweight, overweight, and obesity in the obstetric population during the first trimester of pregnancy between 11 and 13 weeks of gestation [7].

Our study aims to evaluate temporal trends and patterns in pregnant women’s BMI during a 12-year timeframe. In addition to evaluating BMI trends in our population of pregnant women, we also examined BMI’s particularities and variations depending on parity, smoking status, conceiving method, folic acid and multivitamin supplementation, chronic hypertension, and pregestational diabetes. The distribution of and variations in BMI in the Romanian obstetric population have not been reported until now.

## 2. Materials and Methods

We chose 3 stages at which to evaluate the weight of women: before pregnancy, in the first trimester of pregnancy (between 11 and 13 weeks of gestation), and in the second trimester at the moment of the morphological ultrasound (between 18 and 20 weeks of gestation).

We conducted a retrospective cohort study in which we included obstetrically monitored patients between 2010 and 2021. All the women included had singleton pregnancies. The BMIs before pregnancy were obtained by questioning the patients, and for the first and second trimesters of pregnancy by evaluating them. For the first trimester, we used the measurement of the embryo, a CRL (crown–rump length) between 45 and 85 mm (millimeter). For the second trimester, we evaluated our patients’ weights and heights when the pregnancies had a gestational age between 18 and 24 weeks. We also analyzed patients’ age, means of conception, smoking status, and other risk factors (folic acid or vitamin administration, chronic hypertension, or preexisting diabetes). We excluded all duplicate cases, each of our patients being registered in this study only once. The BMI categories were underweight ≤ 18.5; normal weight = 18.5–24.9; overweight = 25–29.9; and obesity = 30 or greater.

### Statistical Analysis

We used the standard classification of BMI: underweight, normal weight, overweight, and obese. The statistics were carried out using SPSS application version 24.

Analysis of variance (ANOVA) was performed to test the differences in mean birth weight and mean gestational age, evaluating BMI variations and trends with the 6 above-mentioned categories (type of conception, smoking habits, parity, COVID-19 pandemic, and folic acid intake). A *p*-value of less than 0.05 was considered significant.

To identify the changes in weight trends, joinpoint regression was estimated for each parameter mentioned above using the Joinpoint Regression Program, Version 4.5.0.1 (Statistical Research and Applications Branch, National Cancer Institute, Rockville, MD, USA).

We used Astraia software version 1.27.1 for pregnancy evaluation and measurements.

## 3. Results

**Figure 1:**
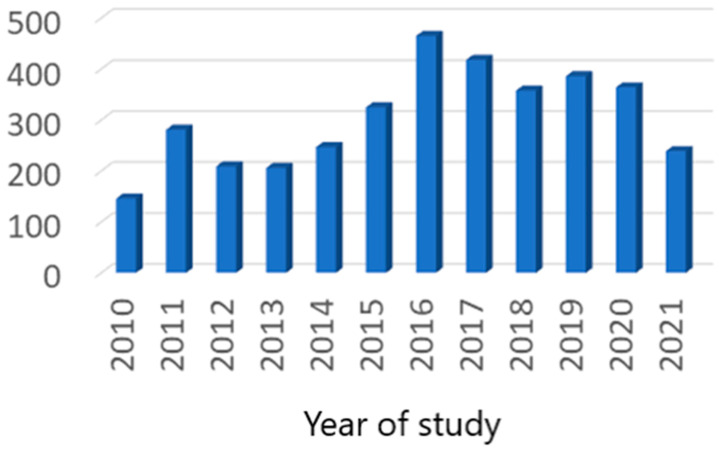
First trimester case distribution.

**Figure 2:**
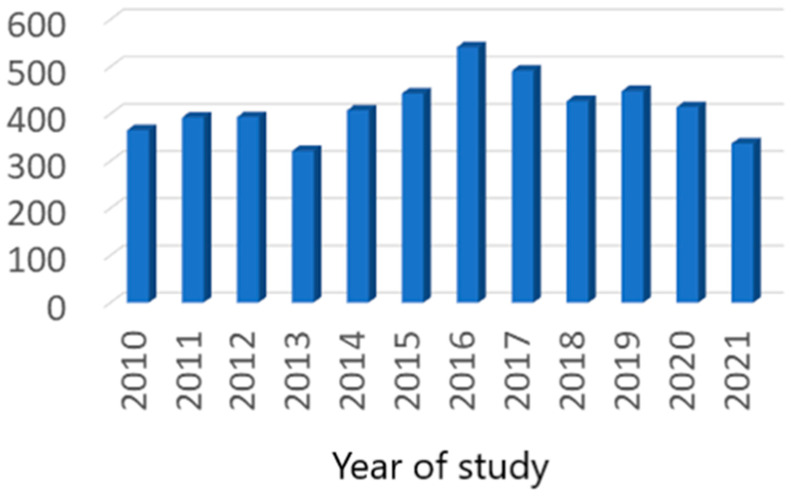
Second trimester case distribution.

All the patients included in our study were Caucasian. After the exclusion of all duplicate cases, we totalized 8.579 patients (3.600 in the first trimester of pregnancy and 4.979 in the second trimester) (Figure 1 and Figure 2).

We noticed a statistically significant difference between the BMIs of nulliparous and multiparous women. This difference was visible before pregnancy and continued throughout the entire gestational period. This aspect led us to separately evaluate multiparous and nulliparous women for each parameter.

### 3.1. Pregestational Results

**Figure 3:**
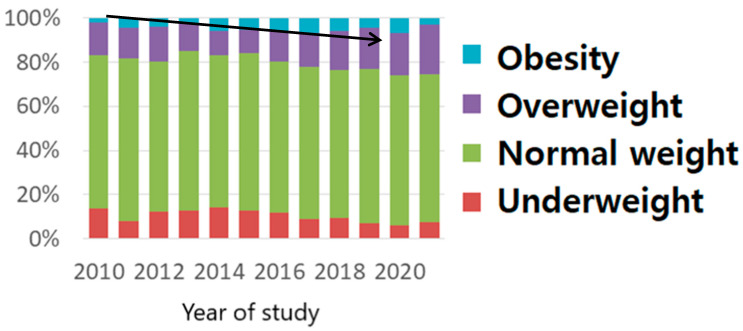
BMI pregestational distribution in nulliparous women (The arrow shows the increasing tendency of obesity as explained in the text).

When we analyzed BMI’s nulliparous pregestational variance, we observed an unequivocal increasing tendency of overweight and obese cases in nulliparous patients throughout our study period. This tendency reached up to 30% in 2020 (Figure 3).

**Figure 4:**
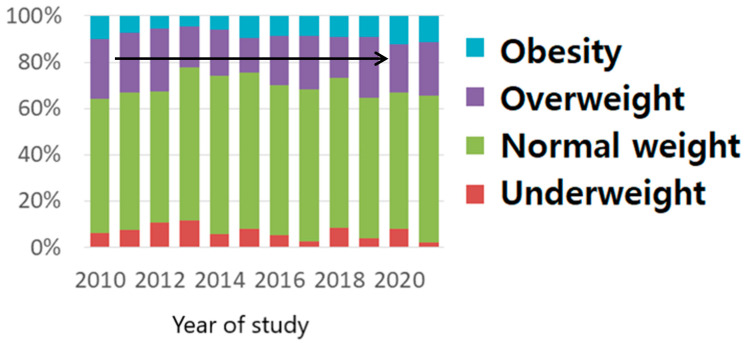
BMI pregestational distribution in multiparous women (The arrow shows the uniform distribution, as explained in the text).

This phenomenon was absent in multiparous women, with the pregestational BMI distribution being practically uniform (Figure 4).

### 3.2. First Trimester Results

We totalized 3.664 cases, but, when we excluded the duplicate ones, we obtained only 3.600 pregnant women who met the inclusion criteria. The mean age of our patients was 30.7 years (±4.8 years), 57.7% of them being nulliparous. When we analyzed the BMI class distribution, we noticed that approximately 29% of the pregnant women included in our study were either overweight (21.7%) or obese (7.7%) at the moment of the first trimester morphology scan. Of these cases, 65% had normal weight, and 5.7% were underweight (Table 1).

**Figure 5:**
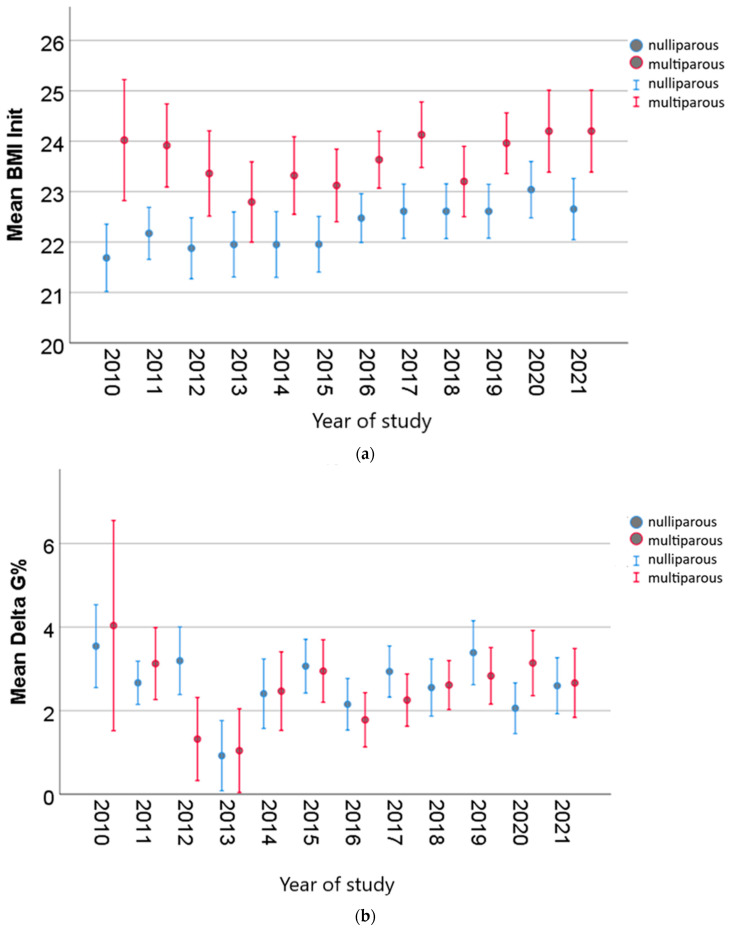
Weight gain (Delta G%) until the second morphological scan for both nulliparous and multiparous women ((**a**), initial BMI; (**b**) mean Delta G%, weight gain in the first trimester).

Despite the existence of differences between nulliparous and multiparous women in the pregestational period, as well as in the first and second trimesters of pregnancy, the weight gain in the first trimester was similar in both categories (Figure 5).

Analyzing the moment of the first trimester morphology scan in nulliparous women, we noticed an increase in the percentage of overweight and obese pregnant women, reaching up to 30% (Figure 3 and Figure 5).

### 3.3. Second Trimester Results

For the second trimester, 4.979 pregnant women met the inclusion criteria. The mean age of our patients was 30.32 years old (±4.84). All of them had singleton pregnancies, 55.1% of them being nulliparous. All the other characteristics are depicted in Table 2. The evaluation was conducted between 18 and 24 weeks of gestation. Almost half of the pregnant women included in our study were overweight or obese (45.7%), with only 53.5% having a normal weight.

When analyzing nulliparous pregnant women during the second trimester of pregnancy, the same increasing tendency for overweight and obese becomes visible. BMI’s distribution in multiparous pregnancies follows the tendency in the pregestational and first trimester time frames by remaining constant.

### 3.4. BMI Joinpoint Regression Analysis

We used joinpoint regression to obtain an objective evaluation of BMI.

**Figure 6:**
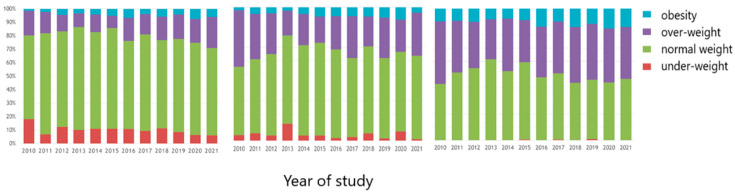
Multiparous weight trend from pregestational period until second trimester of pregnancy.

We detected an annual 0.57 BMI increase in nulliparous women in the first trimester of pregnancy. This increase started in 2013 and reached a maximum mean value in 2020 (Figure 6). The BMI increased annually by 0.18 until 2013.

**Figure 7:**
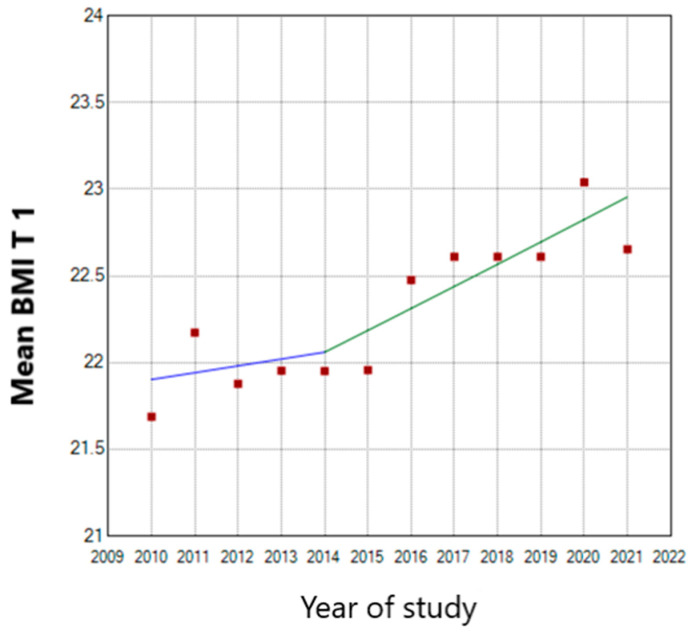
First trimester BMI trend in nulliparous women. (T 1—first trimester of pregnancy; the blue line represents the stationary tendency of the BMI until 2014 and the green line the ascendant tendency of the BMI).

**Figure 8:**
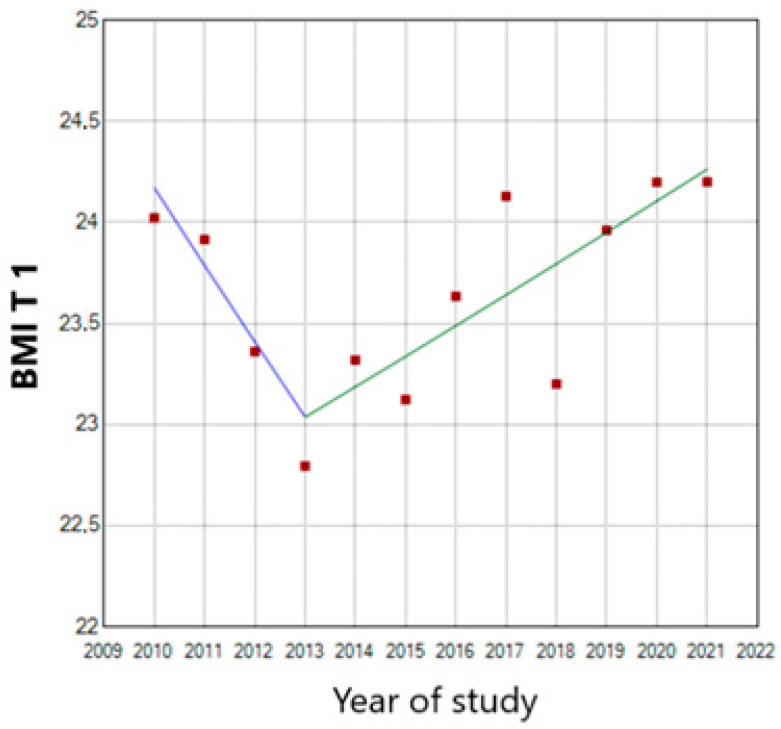
First trimester BMI trend in multiparous women (T 1—first trimester of pregnancy). (the blue line represents the descendent tendency of the BMI until 2014 and the green line the ascendant tendency of the BMI).

When analyzing the first trimester BMI tendency in multiparous women with the same joinpoint regression, we detected a significant annual 0.65 increase starting from 2013. The maximum value was reached in 2020 (Figure 7). During 2010–2013, the annual increase was 1.59 (Figure 8).

**Figure 9:**
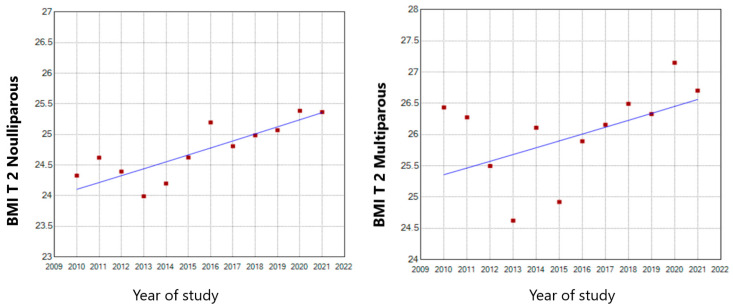
Second trimester BMI trends in nulliparous and multiparous pregnant women (T 2—second trimester of pregnancy).

In the second trimester of pregnancy, both nulliparous and multiparous pregnant women encountered a continuous 0.46 and 0.42 annual BMI increase throughout the whole study period, respectively (Figure 9).

### 3.5. BMI Variation with Other Parameters

#### 3.5.1. Type of Conception

**Figure 10:**
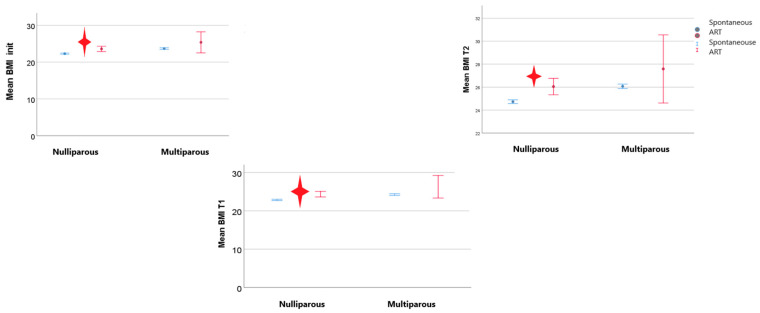
BMI trends depending on the type of conception (BMI_init—pregestational BMI; T1—first trimester of pregnancy; T2—second trimester of pregnancy; the red star indicated statistical significant results).

When evaluating pregestational BMI in nulliparous women depending on the type of conception, our results pointed out a significantly increased pregestational BMI in women with previous ART (assisted reproductive technology) procedures (IVF (in vitro fertilization) or ovarian stimulation) (Figure 10).

**Figure 11:**
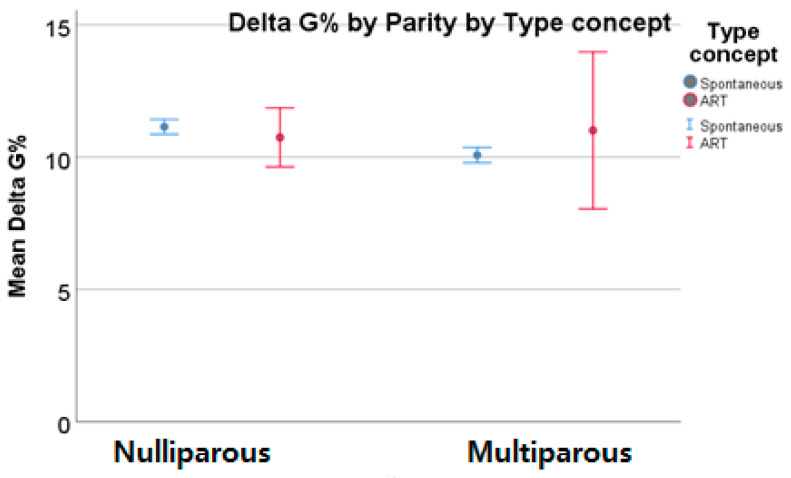
Weight gain depending on the type of conception.

The tendency toward an increased BMI compared with patients with spontaneous conception was maintained throughout the first and the second trimesters of pregnancy. From a weight gain point of view, we detected no differences that concerned the type of conception or parity (Figure 11).

#### 3.5.2. Smoking Habits

**Figure 12:**
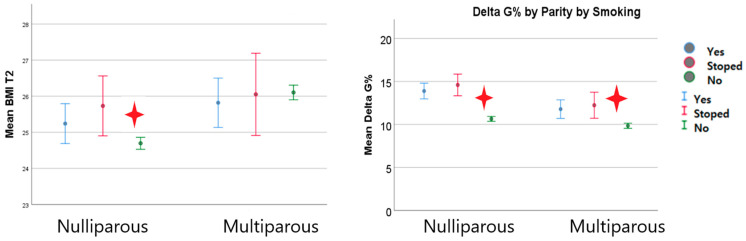
Second trimester BMI in smoker women and their weight gain the red star indicated statistical significant results).

The pregestational and first trimester BMIs of smoking women did not differ so much from that of non-smoking ones, regardless of parity. However, when we analyzed weight in the second trimester of pregnancy, we detected a significantly higher BMI mean value in smoking patients and in patients who quit smoking during pregnancy compared with non-smoking ones. Smoking pregnant women seemed to have higher second trimester weight gain, regardless of parity (Figure 12).

#### 3.5.3. Folic Acid Intake

**Figure 13:**
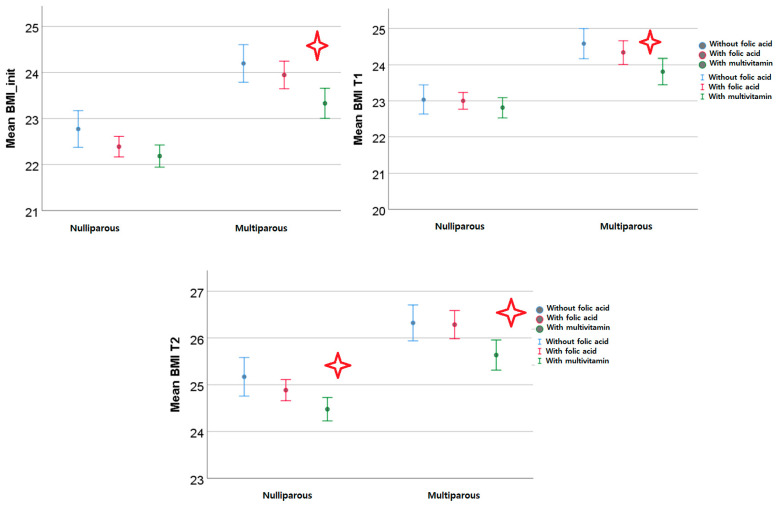
BMI trends depending on folic acid or multivitamin supplementation (BMI_init—pre-gestational BMI; T1—first trimester of pregnancy; and T2—second trimester of pregnancy; the red star indicated statistical significant results).

We analyzed our cohort using folic acid intake, folic acid with multivitamin consumption, or diet supplementation with neither of these two as criteria. The results show no statistically significant pregestational or first or second trimester weight difference in nulliparous women. Instead, there was a statistically significant difference in BMI in the pregestational and first and second trimesters of pregnancy in multiparous women. The women with folic acid supplementation alone had a higher BMI than those with folic acid and multivitamin intake. Multiparous women without folic acid or vitamin consumption had a higher BMI than the other two categories (with folic acid and with vitamin intake), but the difference was not significant. The weight gain was statistically significant in nulliparous women with folic acid diet supplementation in the first trimester and in multiparous women with multivitamin intake during the second trimester until the morphology scan (Figure 13).

#### 3.5.4. Chronic Hypertension

**Figure 14:**
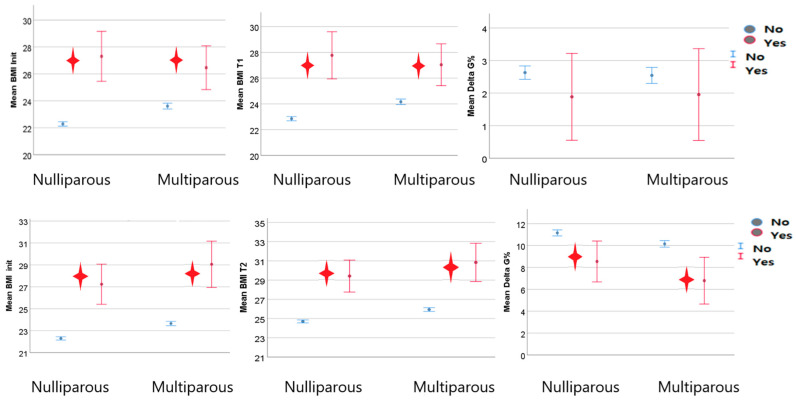
BMI trends in hypertensive women (the red star indicated statistical significant results).

The weight of both nulliparous and multiparous women with chronic hypertension was statistically significantly higher in all three of the time frames in pregestational and the first and second trimesters of pregnancy. However, the weight gain until the second morphological evaluation was statistically significantly lower in women without chronic hypertension (Figure 14).

#### 3.5.5. Pregestational Diabetes

**Figure 15:**
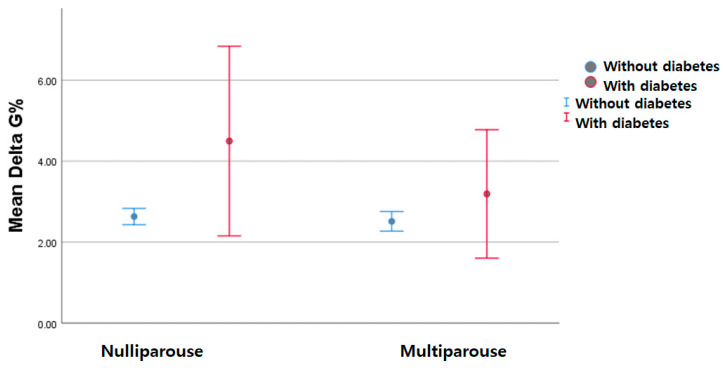
Global weight gain in women with chronic diabetes.

Multiparous diabetic women had statistically significantly higher BMIs than non-diabetic ones in the pre-pregnancy evaluation and the first trimester of pregnancy. The same observation was detected in the second trimester but for both diabetic nulliparous and multiparous pregnant women. The global weight gain did not reveal any statistically significant changes regardless of parity and the pregnancy trimester (Figure 15).

## 4. Discussion

This study allowed us to observe preconception and pregnancy BMI trends over 12 years and their variations depending on parity, the type of conception, smoking status, the COVID-19 pandemic, folic acid or multivitamin intake, chronic hypertension, and diabetes. The large time frame enabled us to obtain solid results and an accurate reflection of our eastern Romanian region. This is the first study conducted in our country that evaluates the BMI of women before pregnancy and during the first two trimesters. Another element that strengthens this study’s value is the analysis of weight trends in women depending on smoking status, the conceiving method, folic acid and multivitamin supplementation, chronic hypertension, and pregestational diabetes.

Maternal nutrition in the pre-pregnancy period and during pregnancy plays an undebated essential role in the development of a fetus. Studies report negative consequences in both underweight and overweight/obesity cases. The intrauterine fetal environment needs to be optimal for the fetus to achieve its full growing and developing potential. Any disruption deeply marks its intrauterine and future childhood and adolescent development due to epigenetic factors. Therefore, we must take care of this to have healthy future generations. Nutritional problems deeply affect our contemporary society with their rising incidence, and it is very hard to implement effective measures to prevent them [1].

As stated in the literature, overweight and obesity have exhibited ascending curves in our women population, while the percentage of normal-BMI women has decreased over time [11,16,17,18]. The main factors involved in this increase are our new sedentary lifestyles, diet modifications characterized by an increased caloric intake of low-cost food, and shifts in our gut microbiomes [16].

Of our patients, 29% were overweight or obese at the moment of the first trimester morphology scan evaluation. The pre-pregnancy percentages were similar to those stated by Wang et al. in their 2021 United States study [19] but lower than the national estimates proposed by Hales et al. in 2020 for the first trimester of pregnancy [20]. However, the percentage increased until the second trimester morphological ultrasound, reaching 45.7%.

Our results are comparable to those stated by Panaitescu et al. in 2019, which evaluated BMI only in the first trimester of pregnancy, concerning the following parameters: maternal age, nulliparous proportion, and means of conception. The proportions of BMI classes are also similar. Parallel to our study, Panaitescu et al.’s results revealed the following first trimester BMI distribution: 6.76% of women were underweight, 66.37% were normal weight, and 26.82% were overweight and obese [7].

Regarding nulliparous women, similar results are depicted in studies from countries neighboring ours, such as Bulgaria and Turkey. The study carried out by Kamburova et al. in Bulgaria [21] states that 23.3% of women are overweight and obese. The percentage from Turkey is closer to ours, with 27.2% of women being obese and overweight in the first trimester of pregnancy [22]. Concerning the second trimester of pregnancy, the rates of overweight and obese women exceed 40%, as Fleming et al. in their study state to be the situation in most developing countries.

The peak for nulliparous women was reached in 2020, with a 30% increase compared with multiparous women, who exhibited a constant trend during the 12 years of study, but with a value of overweight and obesity that exceeded 30% [23,24]. As stated in Reynolds et al.’s [17] and McKeating et al.’s studies [23], multiparity is a risk factor for overweight and obesity. Our study’s results also strengthen this idea. Studies have evaluated interpregnancy weight gain and maternal and fetal outcomes. The percentage of overweight and obese women was high and had a constant trend during the 12 years of our study. This reveals an important interpregnancy weight gain in our multiparous women. This increase in women’s weight, which takes place from one pregnancy to another, has a negative influence on both mother and fetal outcomes. It is associated with pregnancy hypertensive disorders, diabetes, LGA fetuses, stillbirths, and C-section births [17,23,24,25] (McKeating). Our study also indicates that obese women are more prone to higher weight gain during pregnancy, in agreement with Rasmussen et al. [25].

We also detected a trend to postpone the first pregnancy, with the mean age for nulliparous women in our study being 30.69 ± 4.81. This tendency to postpone pregnancy is also visible in Panaitescu et al.’s study, strengthening the idea that this is a generalized phenomenon in our country [7].

Our study might underestimate the proportion of overweight and obesity in women who fail in spontaneous conception because this systemic inflammatory pathology is frequently associated with infertility, miscarriage, and congenital malformations [26,27]. We only included women who succeeded in conceiving via ovarian stimulation or IVF in our study.

When analyzing overweight and obesity depending on the other variables in our study, the results regarding smoking women were consistent with those of McKeating et al. They found no difference between smoking and non-smoking women concerning the percentage of overweight and obesity in the pre-pregnancy and first trimester time frames. However, in the second trimester, we observed increased weight gain, with higher proportions of overweight and obese pregnant women in smokers and those who quit smoking during pregnancy.

As stated in our study, Akter et al. found an increase in the percentage of overweight people during the pandemic mainly due to a sedentary lifestyle [28]. This aspect was also sustained by Restrepo et al. in their United States study [29].

Mlodzik-Czyzewska et al.’s case control study underlines that low folate intake and low serum folate values are associated with overweight and obesity [30]. This trend is also visible in our study, especially in multiparous women, such that multiparous women without any supplementation had higher BMI values than those with vitamin or folic acid supplementation. Many studies reveal the weight gain protector effect of a mother’s folic acid intake on their children [31]. These results underline the importance of folic acid supplementation, either alone or in association with a multivitamin, in the pregestational period in women who desire to conceive. Supplementation with folic acid has two major benefits for both the mother and the child concerning its protective weight gain effect, the counterbalancing of which extends throughout generations.

Being overweight and obese before pregnancy implies a multitude of well-known complications. A wide range of such complications, such as preeclampsia, admission to the neonatal intensive care unit, fetal growth pathology, premature birth, C-section delivery, and superimposed preeclampsia, negatively influence a woman with chronic hypertension throughout her pregnancy’s evolution. As stated in the literature, chronic hypertensive disorder is directly associated with overweight and obesity [32]. Our study reinforces these aspects. Our chronic hypertensive cohort of women had higher BMIs than the rest. However, the interesting aspect that we noticed was that the weight gain until the second morphological evaluation was statistically significantly lower in women without chronic hypertension.

The literature proves a direct relationship between the increase in obesity rates and pre-pregnancy diabetes [33]. These two pathologies are highly interconnected. This is sustained by our results, which obtained a higher BMI in diabetic women compared with non-diabetic ones and a statistically significantly increased BMI in multiparous women. However, the weight gain during pregnancy until the second morphological scan was not statistically significantly higher in women with chronic diabetes than in those without.

The assessment of pre-pregnancy BMI becomes very important when considering that the major impact on maternal and fetal outcomes is consistent with overweight and obesity before pregnancy, and weight gain during pregnancy has a reduced influence [34]. Dietary and other interventions aiming to improve maternal and fetal outcomes ought to concentrate on the pre-pregnancy period to obtain a significant result, underlining the importance of our study, which evaluated women’s pre-pregnancy BMIs. This also adds value to our study, which succeeded in revealing women’s BMI class distribution before and during pregnancy.

Another important aspect is that the BMI class distribution in the population varies with race, ethnicity, maternal age, and a country’s economic status. The population in our study was homogenous. All the women included were Caucasian. The proportion of pre-pregnancy overweight and obese women was far smaller than the 46% described by Fisher et al. in their United States study (Fisher). Moreover, of the three times that BMI was evaluated in our study, the pre-pregnancy value was assessed by interviewing the patient the first time only, and the second two values were obtained via a direct evaluation of each pregnant woman by healthcare professionals in a limited timeframe. Another of our study’s strengths resides in the 12-year period we took into consideration.

One of the limitations of our study is that it was exclusive to women who could not conceive, so the rate of pre-pregnancy overweight and obesity was underestimated. Furthermore, our study was limited to only one center in the private sector.

## 5. Conclusions

Obesity is, and will remain for a long time, one of the major burdens of the world’s healthcare systems. It has gained prevalence over time as an epidemic. The health complications and overall impact on maternal and fetal outcomes surpass a specific moment in time, extending to future generations due to epigenetics.

Our article describes the trends of obesity and overweight in our middle-income country, in which this pathology is undergoing continuous growth, negatively influencing our reproductive-aged women and future generations.

Overweight and obese patients require expensive individualized health management.

We need to make a sustained effort to stop this trend and succeed in improving the health of our people.

## Figures and Tables

**Table 1:** First trimester pregnant women characteristics.

First Trimester Characteristics		
Mean age		30.69 ± 4.81
Gestational age		12.46 ± 0.65
CRL		61.92 ± 11.72
Nulliparous		57.7%
Folic acid	Without folic acid supplementation	20.3%
	Folic acid	47.6%
	Folic acid with multivitamins	32.1%
Chronic hypertension		1.9%
Diabetes		0.7%
Type of conception	Spontaneous	95.5%
	ART	4.3%
Smoking	Yes	8.7%
	No	86.7%
Initial BMI		22.93 ± 4.06
First trimester BMI		23.49 ± 4.08
BMI class	Underweight	5.7%
	Normal weight	65%
	Overweight	21.7%
	Obese	7.7%
Delta G% (weight gain)		2.57 ± 4.71

**Table 2:** Second trimester pregnant women’s characteristics.

Second Trimester Pregnant Women’s Characteristics		
Age		30.32 ± 4.84
Gestational age		22.09 ± 1.31
Nulliparous		55.1%
Folic acid	Without folic acid supplementation	20.6%
	Folic acid	45.3%
	Folic acid with multivitamins	34.1%
Chronic hypertension		1.9%
Diabetes		0.6%
Type of conception	Spontaneous	97.1%
	ART	2.9%
Smoking	Yes	8.9%
	No	86.9%
Initial BMI		22.96 ± 4.13
First trimester BMI		25.32 ± 4.16
BMI class	Underweight	0.8%
	Normal weight	53.5%
	Overweight	32.3%
	Obese	13.4%
Delta G% (weight gain)		10.7 ± 6.68

## Data Availability

Data are contained within the article.

## References

[1] Ratnasiri A.W.G., Lee H.C., Lakshminrusimha S., Parry S.S., Arief V.N., DeLacy I.H., Yang J.S., DiLibero R.J., Logan J., Basford K.E. (2019). Trends in maternal prepregnancy body mass index (BMI) and its association with birth and maternal outcomes in California, 2007-2016: A retrospective cohort study. PLoS ONE.

[2] Paljk I., Verdenik I., Blickstein I., Tul N. (2021). Maternal BMI and weight gain in singleton pregnancies: Has something changed in the last decade?. J. Matern. Fetal Neonatal Med..

[3] Martin B., Sacks D.A. (2018). The global burden of hyperglycemia in pregnancy—Trends from studies in the last decade. Diabetes Res. Clin. Pract..

[4] Morais S.S., Ide M., Morgan A.M., Surita F.G. (2017). A novel body mass index reference range—An observational study. Clinics.

[5] Morgan K.L., Rahman M.A., Macey S., Atkinson M.D., Hill R.A., Khanom A., Paranjothy S., Husain M.J., Brophy S.T. (2014). Obesity in pregnancy: A retrospective prevalence-based study on health service utilisation and costs on the NHS. BMJ Open.

[6] Poston L. (2017). Obesity in pregnancy; Where are we, where should we go?. Midwifery.

[7] Panaitescu A.M., Rotaru D., Ban I., Peltecu G., Zagrean A.M. (2019). The prevalence of underweight, overweight and obesity in a Romanian population in the first trimester of pregnancy—Clinical implications. Acta Endocrinol. (Buchar.).

[8] Branum A.M., Kirmeyer S.E., Gregory E.C. (2016). Prepregnancy body mass index by maternal characteristics and state: Data from the birth certificate, 2014. Natl. Vital Stat. Rep..

[9] World Health Organization (2000). Obesity: Preventing and managing the global epidemic. Report of a WHO consultation. World Health Organ. Tech. Rep. Ser..

[10] Maxwell C., Gaudet L., Cassir G., Nowik C., McLeod N.L., Jacob C.É., Walker M. (2019). Guideline No. 391-Pregnancy and Maternal Obesity Part 1: Pre-conception and Prenatal Care. J. Obstet. Gynaecol. Can..

[11] Deputy N.P., Dub B., Sharma A.J. (2018). Prevalence and Trends in Prepregnancy Normal Weight—48 States, New York City, and District of Columbia, 2011–2015. MMWR Morb. Mortal. Wkly. Rep..

[12] Horta B.L., Barros F.C., Lima N.P., Assunção M.C.F., Santos I.S., Domingues M.R., Victora C.G., Pelotas Cohorts Study Group (2019). Maternal anthropometry: Trends and inequalities in four population-based birth cohorts in Pelotas, Brazil, 1982–2015. Int. J. Epidemiol..

[13] Scott-Pillai R., Spence D., Cardwell C., Hunter A., Holmes V. (2013). The impact of body mass index on maternal and neonatal outcomes: A retrospective study in a UK obstetric population, 2004–2011. BJOG.

[14] Godfrey K.M., Costello P.M., Lillycrop K.A. (2015). The developmental environment, epigenetic biomarkers and long-term health. J. Dev. Orig. Health Dis..

[15] Poston L., Harthoorn L.F., Van Der Beek E.M., Contributors to the ILSI Europe Workshop (2011). Obesity in pregnancy: Implications for the mother and lifelong health of the child. A consensus statement. Pediatr. Res..

[16] Ng M., Fleming T., Robinson M., Thomson B., Graetz N., Margono C., Mullany E.C., Biryukov S., Abbafati C., Abera S.F. (2014). Global, regional, and national prevalence of overweight and obesity in children and adults during 1980–2013: A systematic analysis for the Global Burden of Disease Study 2013. Lancet.

[17] Reynolds C.M.E., Egan B., McMahon L., O’Malley E.G., Sheehan S.R., Turner M.J. (2019). Maternal obesity trends in a large Irish university hospital. Eur. J. Obstet. Gynecol. Reprod. Biol..

[18] Fisher S.C., Kim S.Y., Sharma A.J., Rochat R., Morrow B. (2013). Is obesity still increasing among pregnant women? Prepregnancy obesity trends in 20 states, 2003–2009. Prev. Med..

[19] Wang M.C., Freaney P.M., Perak A.M., Greenland P., Lloyd-Jones D.M., Grobman W.A., Khan S.S. (2021). Trends in Prepregnancy Obesity and Association with Adverse Pregnancy Outcomes in the United States, 2013 to 2018. J. Am. Heart Assoc..

[20] Hales C.M., Carroll M.D., Fryar C.D., Ogden C.L. (2020). Prevalence of obesity and severe obesity among adults: United States, 2017–2018. NCHS Data Brief.

[21] Kamburova M.S., Hristova P.A., Georgieva S.L., Khan A. (2015). Adverse effects of maternal age, weight and smoking during pregnancy in Pleven, Bulgaria. South Eastern Eur. J. Public Health (SEEJPH).

[22] Daşıkan Z. (2015). Weight Gain in Pregnancy: Do Pregnant Women Receive Correct Weight Gain Recommendation in Prenatal Care?. Turk. Klin. Jinekoloji Obstet..

[23] McKeating A., Maguire P.J., Daly N., Farren M., McMahon L., Turner M.J. (2015). Trends in maternal obesity in a large university hospital 2009–2013. Acta Obstet. Gynecol. Scand..

[24] Oteng-Ntim E., Mononen S., Sawicki O., Seed P.T., Bick D., Poston L. (2018). Interpregnancy weight change and adverse pregnancy outcomes: A systematic review and meta-analysis. BMJ Open.

[25] Rasmussen K.M., Yaktine A.L. (2009). Weight Gain during Pregnancy: Reexamining the Guidelines Institute of Medicine and National Research Council Committee to Reexamine IOM Pregnancy Weight Guidelines.

[26] Catalano P.M., Shankar K. (2017). Obesity and pregnancy: Mechanisms of short term and long term adverse consequences for mother and child. BMJ.

[27] Langley-Evans S.C., Pearce J., Ellis S. (2022). Overweight, obesity and excessive weight gain in pregnancy as risk factors for adverse pregnancy outcomes: A narrative review. J. Hum. Nutr. Diet..

[28] Akter T., Zeba Z., Hosen I., Al-Mamun F., Mamun M.A. (2022). Impact of the COVID-19 pandemic on BMI: Its changes in relation to socio-demographic and physical activity patterns based on a short period. PLoS ONE.

[29] Restrepo B.J. (2022). Obesity Prevalence Among U.S. Adults During the COVID-19 Pandemic. Am. J. Prev. Med..

[30] Mlodzik-Czyzewska M.A., Malinowska A.M., Chmurzynska A. (2020). Low folate intake and serum levels are associated with higher body mass index and abdominal fat accumulation: A case control study. Nutr. J..

[31] Wang G., DiBari J., Bind E., Steffens A.M., Mukherjee J., Azuine R.E., Singh G.K., Hong X., Ji Y., Ji H. (2019). Association Between Maternal Exposure to Lead, Maternal Folate Status, and Intergenerational Risk of Childhood Overweight and Obesity. JAMA Netw. Open.

[32] Badon S.E., Dublin S., Nance N., Hedderson M.M., Neugebauer R., Easterling T., Cheetham T.C., Chen L., Holt V.L., Avalos L.A. (2021). Gestational weight gain and adverse pregnancy outcomes by pre-pregnancy BMI category in women with chronic hypertension: A cohort study. Pregnancy Hypertens..

[33] Fadl H.E., Simmons D. (2016). Trends in diabetes in pregnancy in Sweden 1998–2012using. BMJ Open Diabetes Res. Care.

[34] Zhang J., Zhang R., Chi J., Li Y., Bai W. (2023). Pre-pregnancy body mass index has greater influence on newborn weight and perinatal outcome than weight control during pregnancy in obese women. Arch. Public Health.

